# Abplatin^(IV)^ inhibited tumor growth on a patient derived cancer model of hepatocellular carcinoma and its comparative multi-omics study with cisplatin

**DOI:** 10.1186/s12951-022-01465-y

**Published:** 2022-06-03

**Authors:** Xing Li, Lingpu Zhang, Tuo Li, Shumu Li, Wenjing Wu, Lingyu Zhao, Peng Xie, Jinqi Yang, Peipei Li, Yangyang Zhang, Haihua Xiao, Yingjie Yu, Zhenwen Zhao

**Affiliations:** 1 Beijing National Laboratory for Molecular Sciences, CAS Research/Education Center for Excellence in Molecular Sciences, Institute of Chemistry Chinese Academy of Sciences, Beijing Mass Spectrum Center, Beijing, 100190 China; 2 College of Life Science and Technology; State Key Laboratory of Organic-Inorganic Composites, Beijing University of Chemical Technology, Beijing, 100029 China; 3 Department of Nuclear Medicine, Peking Union Medical College Hospital, Chinese Academy of Medical Science & Peking Union Medical College, Beijing, 100730 China; 4 Department of Orthopedics, The Second Xiangya Hospital, Central South University, Changsha, 410011 Hunan China; 5 Graduate School, University of Chinese Academy of Sciences, Beijing, 100049 China

**Keywords:** Pt(IV) drugs, Abplatin^(IV)^, Multi-omics, Nanoparticles, Cisplatin

## Abstract

**Background:**

Cisplatin, the alkylating agent of platinum(II) (Pt(II)), is the most common antitumor drug in clinic; however, it has many side effects, therefore it is higly desired to develop low toxicity platinum(IV) (Pt(IV)) drugs. Multi-omics analysis, as a powerful tool, has been frequently employed for the mechanism study of a certain therapy at the molecular level, which might be helpful for elucidating the mechanism of platinum drugs and facilitating their clinical application.

**Methods:**

Strating form cisplatin, a hydrophobic Pt(IV) prodrug (CisPt(IV)) with two hydrophobic aliphatic chains was synthesized, and further encapsulated with a drug carrier, human serum albumin (HSA), to form nanoparticles, namely AbPlatin^(IV)^. The anticancer effect of AbPlatin^(IV)^ was investigated in vitro and in vivo. Moreover, transcriptomics, metabolomics and lipidomics were performed to explore the mechanism of AbPlatin(IV).

**Results:**

Compared with cisplatin, Abplatin^(IV)^ exhibited better tumor-targeting effect and greater tumor inhibition rate. Lipidomics study showed that Abplatin^(IV)^ might induce the changes of BEL-7404 cell membrane, and cause the disorder of glycerophospholipids and sphingolipids. In addition, transcriptomics and metabolomics study showed that Abplatin^(IV)^ significantly disturbed the purine metabolism pathway.

**Conclusions:**

This research highlighted the development of Abplatin^(IV)^ and the use of multi-omics for the mechanism elucidation of prodrug, which is the key to the clinical translation of prodrug.

**Graphical Abstract:**

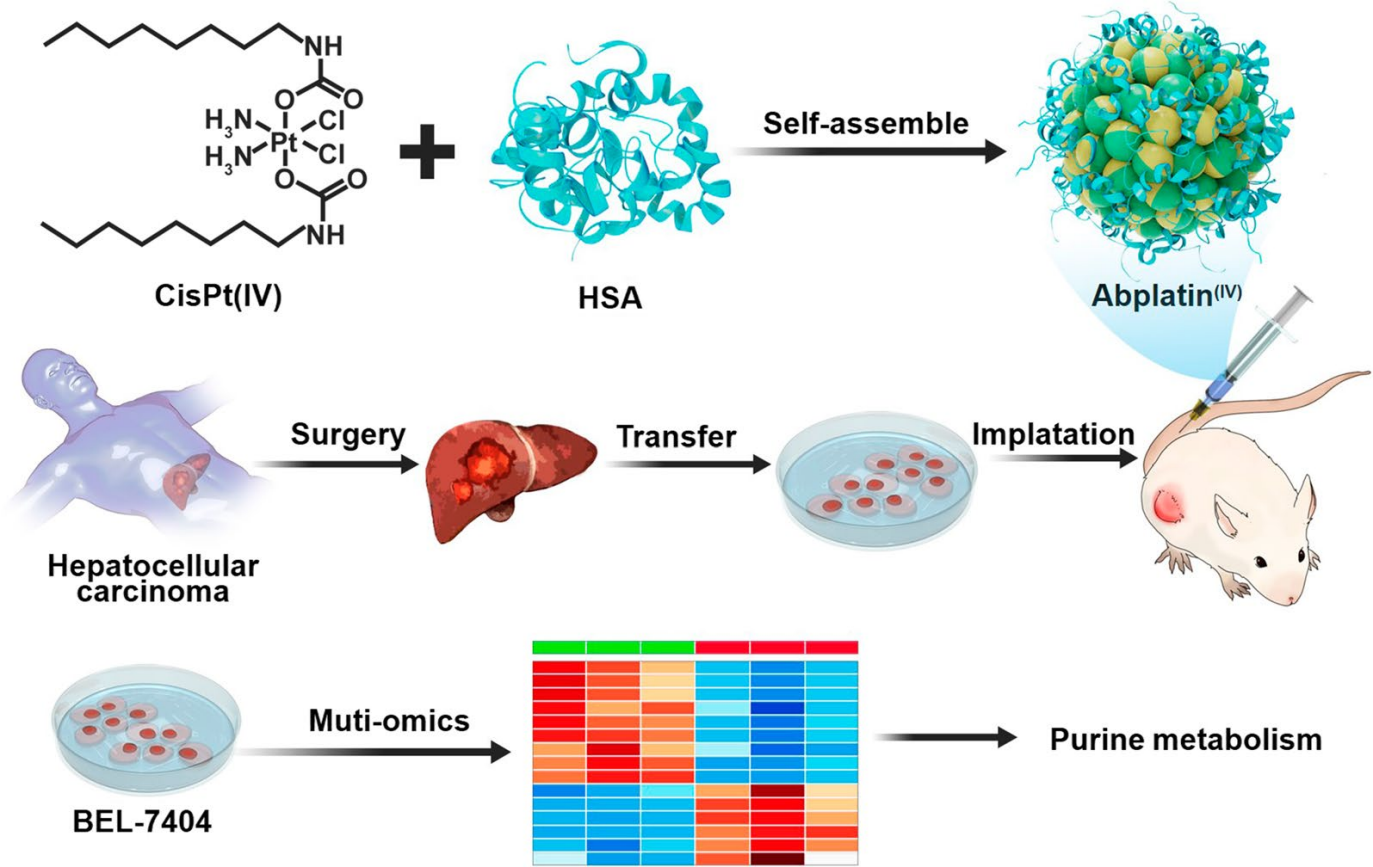

**Supplementary Information:**

The online version contains supplementary material available at 10.1186/s12951-022-01465-y.

## Background

Cisplatin, the alkylating agent of platinum(II) (Pt(II)), is the most common antitumor drug in clinic. It induces cell apoptosis by cross-linking with DNA [1–3]. The major obstacles of cisplatin are its side effects as well as evolution of drug resistance [1, 4]. Therefore, in recent years, numerous platinum(IV) (Pt(IV)) prodrugs have been prepared [5–8]. In addition, to further enhance the targeting efficiency of Pt(IV) prodrugs and reduce their toxicity, people have also developed various drug carriers such as polymers, lipids, proteins, and peptides to physically encapsulate or chemically conjugate with these Pt(IV) prodrugs, resulting in thousands of Pt(IV)-based drug delivery systems (DDS) [9–12]. Due to the enhanced permeability and retention (EPR) effect, nano drugs can effectively accumulate at tumor tissues, hence leading to lower toxicity and better efficacy [13]. Zeng et al*.* found that HSA with platinum ions in the blood of patients could form nanoparticles after intravenous cisplatin administration, and these nanoparticles could successfully accumulated at the tumor region [14]. Hence, utilizing HSA to encapsulate Pt(IV) prodrug coud be an efficient strategy for enhanced chemotherapy, which would extend the application of platinum-based chemotherapy.

It is generally believed that after Pt(IV)-based DDS enter cancer cells, Pt(IV) prodrugs are reduced to Pt(II) by intracellular reducing agents like glutathione (GSH) and ascorbic acid. Then, the released Pt(II) drugs bind with DNA to activate cell killing effect [15–18]. However, there are different views that Pt(IV) prodrugs themselves can interact with DNA to exert the anticancer effect, making the mechanism of Pt(IV) prodrugs and their DDS more blurred. In a word, up to now, Although thousands of Pt(IV) prodrugs and their DDS have been developed, and some of them showed excellent clinical translational prospect and even entered clinical research, the mechanism of Pt(IV) prodrugs and their DDS at the molecular level remains unknown, which hampers their further clinical translation. Multi-omics analysis can help one to understand the interactions between a variety of substances in biological system, which is of great value in exploring cancer pathogenesis, screening treatment targets, and discovering biomarkers for early diagnosis, drug sensitivity prediction, and cancer prognosis [19–23]. The systematic analysis of genes, lipids, and small molecule metabolites could help to deepen the understanding of the mechanism of Pt(IV) prodrugs.

**Scheme 1:**
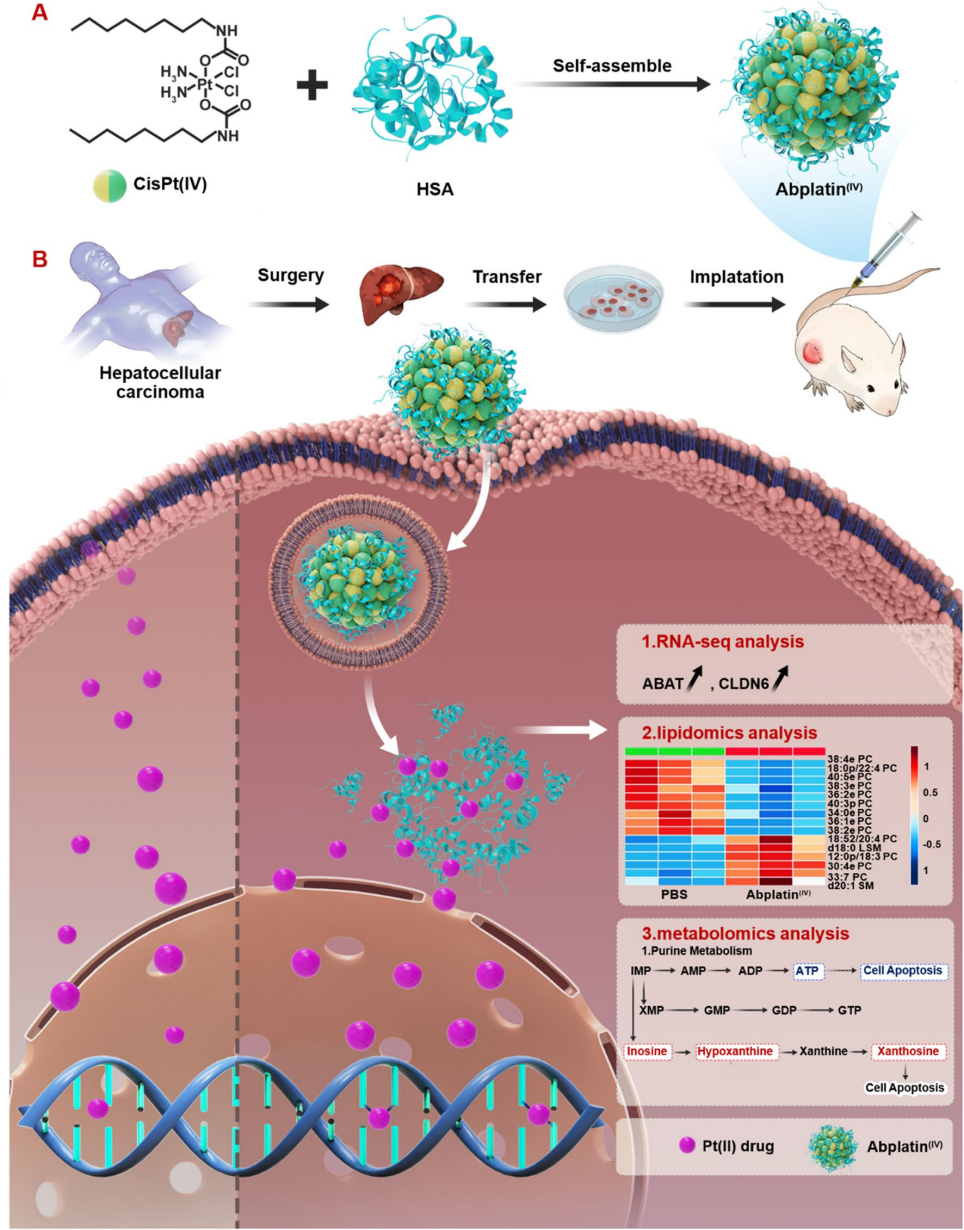
Schematic illustration showing Abplatin^(IV)^ inhibited the tumor growth on a patient-derived tumor xenograft model of hepatocellular carcinoma (PDX^HCC^) and its comparative multi-omics study with cisplatin. **A** Abplatin^(IV)^ was prepared by the physical encapsulation of a hydrophobic CisPt(IV) with HSA. **B** Subsequently, Abplatin^(IV)^ was intraveneously (*i.v.*) injected into the mice bearing PDX^HCC^. Abplatin^(IV)^ could be accumulated at the tumor site via blood circulation and internalized by the cancer cells via endocytosis. **C** The possible mechanism of Cisplatin (Left panel) and Abplatin^(IV)^ (Right panel). The Pt(II) in cisplatin might directly bind to DNA to cause cell apoptosis. However, Abplatin^(IV)^ was believed to be reduced by intracellular reducing agents such as glutathione(GSH) and ascorbic acid to release Pt(II) for DNA binding. Transcriptomics and metabolomics study on the mechanism of Abplatin^(IV)^ revealed Abplatin^(IV)^ significantly disturbed the purine metabolism pathway. Up-regulated and down-regulated metabolites were represented in red and blue, respectively. Lipidomics study showed that Abplatin^(IV)^ induced the disorder of glycerophospholipids and sphingolipids

Herein, starting from cisplatin, we firstly prepared a hydrophobic Pt(IV) prodrug (CisPt(IV)), which is characterized by the presence of two apliphatic chains in the axial position. To further deliver CisPt(IV), HSA was selected to encapsulate this prodrug to form nanoparticles, namely AbPlatin^(IV)^ (Scheme 1A). AbPlatin^(IV)^ was supposed to release Pt(II) after it entered the cancer cells via chemical reduction. AbPlatin^(IV)^ had strong killing ability to cancer cells, even cisplatin-resistant cells. We had shown here on a hepatocellular carcinoma patient-derived tumor xenograft model (PDX^HCC^), AbPlatin^(IV)^ also exhibited a promising anti-tumor effect with excellent tumor targeting and low toxic side effects (Scheme 1B). To understand the mechanism of AbPlatin^(IV)^, we further systematically investigated the effects of AbPlatin^(IV)^ compared with cisplatin on the transcriptomics, metabolomics, and lipidomics. It was found that Abplatin^(IV)^ significantly affected purine metabolism pathway. Therein, ATP was down-regulated, and xanthosine and hypoxanthine were up-regulated in cells treated with Abplatin^(IV)^, which might be contributed to cell apoptosis. Besides, it was found that Abplatin^(IV)^ affected more genes expression than cisplatin, like that ABAT and CLDN6 were significantly up-regulated. We also found carnitine was down-regulated, and arginine content was up-regulated. This work highlighted the use of multi-omics for the mechanism study of Abplatin^(IV)^.

## Results and discussion

### Preparation and characterization of Abplatin^(IV)^

**Fig. 1:**
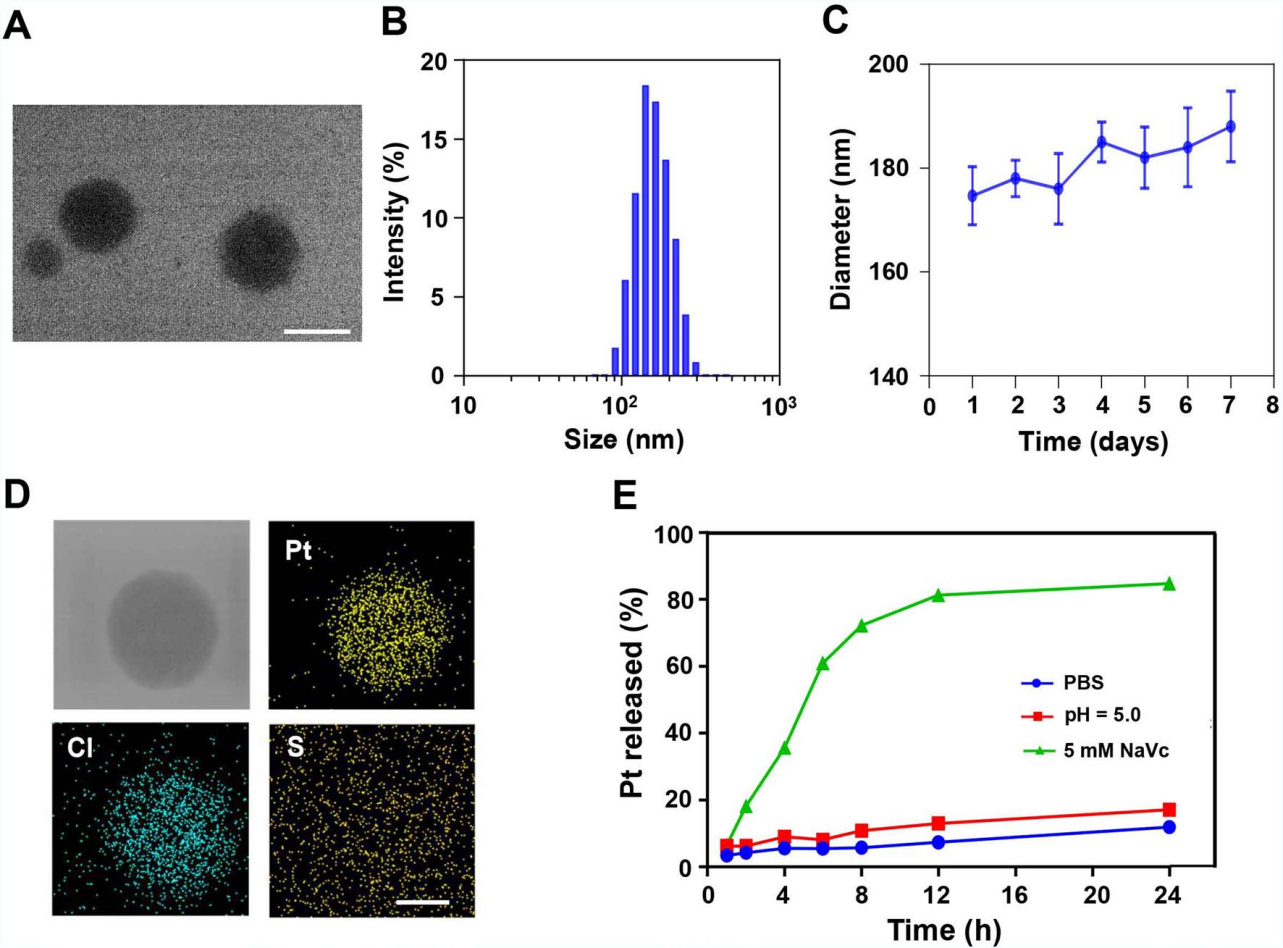
Characterization of Abplatin^(IV)^. **A** Representative transmission electron microscope (TEM) image of Abplatin^(IV)^, scale bar = 200 nm. **B** Hydrodynamic diameter of Abplatin^(IV)^ by dynamic light scattering (DLS). **C** Particle size of Abplatin^(IV)^ following incubation in PBS for 7 days. **D** Element mapping of Abplatin^(IV)^ by scanning transmission electron microscope (STEM), scale bar = 100 nm. **E** Representative drug release profile of Abplatin^(IV)^ under various conditions

CisPt(IV) with two aliphatic chains was synthesized as previously described (Additional file 1: Fig. S1) and characterized with ESI–MS (Additional file 1: Fig. S2) [24]. HSA is the most abundant protein in the human bloodstream [25, 26], which is widely used as drug carriers [27–29]. Therefore, Abplatin^(IV)^ was prepared by encapsulation of CisPt(IV) with HSA. Compared with CisPt(IV), Abplatin^(IV)^ had better water solubility, which was conducive to the biological application. Transmission electron microscopy (TEM) and dynamic light scattering (DLS) demonstrated that Abplatin^(IV)^ was in spherical structure with a diameter at 174.7 nm at a feed mass ratio of 1/10 (Pt to HSA) (Fig. 1A and B). Moreover, Abplatin^(IV)^ exhibited excellent stability within 7 days with limited size variations (Fig. 1C). Element mapping mappings of Abplatin^(IV)^ via scanning transmission electron microscope (STEM) revealed that Abplatin^(IV)^ contained Cl and Pt, and X-ray photoelectron spectroscopy (XPS) showed that CisPt(IV) did not contain sulfur element while Abplatin^(IV)^ contained sulfur element (Additional file 1: Fig. S3), together confirming that CisPt(IV) was successfully encapsulated into the nanoparticles by HSA (Fig. 1D). Abplatin^(IV)^ was believed to be reduced by intracellular reducing agents such as glutathione (GSH) and ascorbic acid to release Pt(II) for DNA binding [15, 16, 30]. The results showed that within 12 h, 80%, 10% and 8% of Pt drugs were released in sodium ascorbate (NaVc) aqueous solution, acetate buffer solution, and PBS, respectively, indicating Pt drugs could be rapidly released under the reductive environment (Fig. 1E).

### In vitro anticancer efficacy of Abplatin^(IV)^

**Fig. 2:**
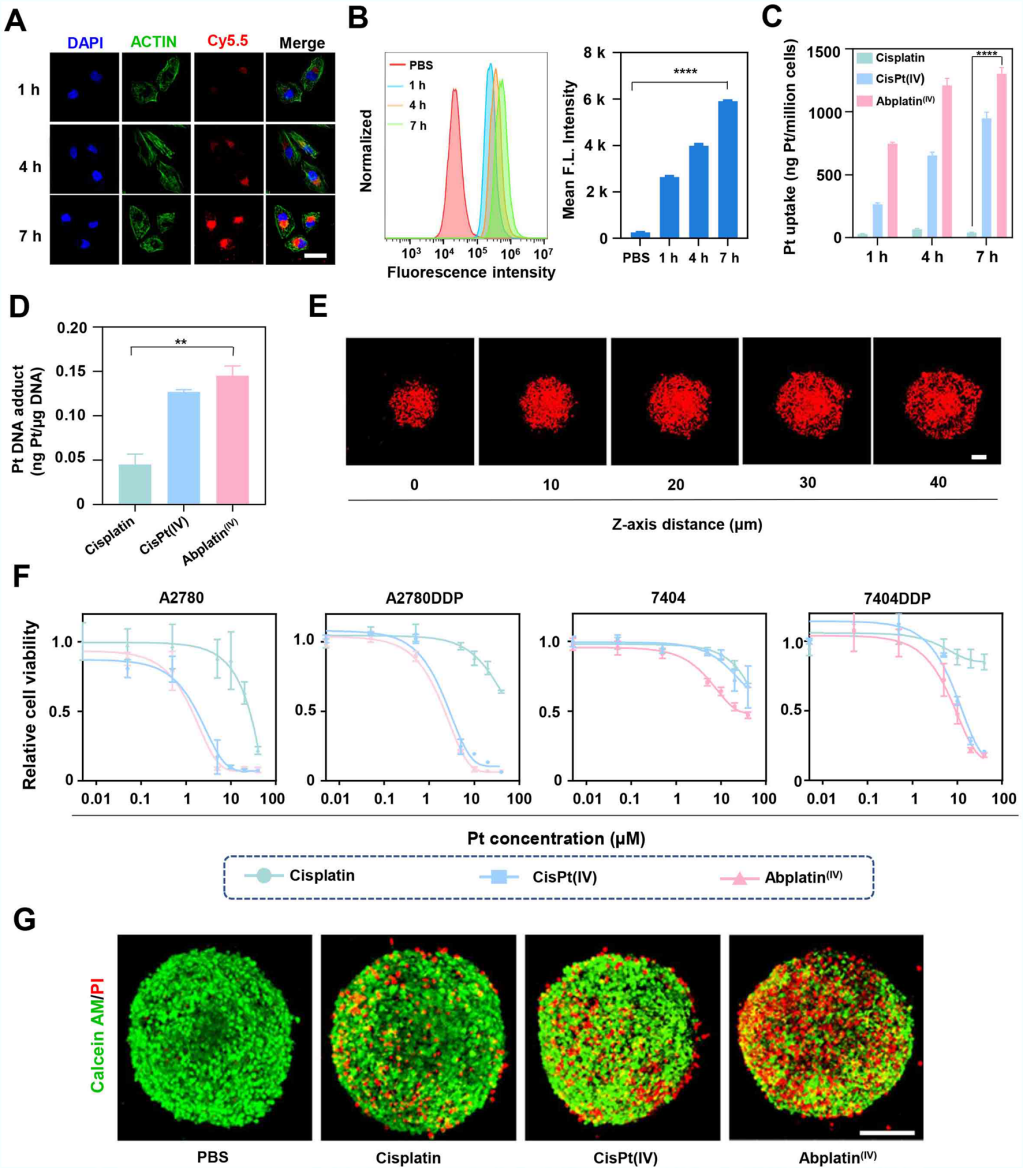
Intracellular uptake and in vitro anticancer efficacy of Abplatin^(IV)^. **A** Intracellular uptake of Abplatin^(IV)^@Cy5.5 by BEL-7404 cells via confocal laser scanning microscope (CLSM), scale bar = 40 µm. **B** Semi-quantitative study of the intracellular uptake of Abplatin^(IV)^@Cy5.5 *via* flow cytometry. **C** Intracellular Pt uptake of the cells treated with various drugs (10 μM Pt) for 1, 4, and 7 h respectively via ICP-MS. **D** Pt-DNA adducts of cells treated with cisplatin, CisPt(IV), and Abplatin^(IV)^ via ICP-MS. **E** Fluorescence distribution of Abplatin^(IV)^@Cy5.5 at 12 h in different focal planes of 3D tumor sphere, scale bar = 100 µm. **F** In vitro anticancer activity of cisplatin, CisPt(IV) and Abplatin^(IV)^. **G** Cell apoptosis in 3D tumor sphere induced by various drugs (10 µM) in 3D tumor spheres at 24 h, scale bar = 200 µm

Abplatin^(IV)^ was supposed to be endocytosed by BEL-7404 cancer cells. To prove this process, Abplatin^(IV)^ was firstly labelled with Cy5.5 (Abplatin^(IV)^@Cy5.5) and observed by confocal laser scanning microscope (CLSM). The results demonstrated that the red fluorescence in the BEL-7404 cells increased with longer incubation time (Fig. 2A). Further semi-quantification via flow cytometry showed that the uptake of Abplatin^(IV)^@Cy5.5 at 7 h was 2.24 times than that at 1 h (Fig. 2B). The above results together demonstrated that the Abplatin^(IV)^ could effectively enter into the cells. To futher explore the tumor penetration of Abplatin^(IV)^, a 3D cell sphere was established. It showed all focal planes were full of red, indicating that Abplatin^(IV)^ could effectively penetrate into tumor spheroids (Fig. 2E).

There are numerous Pt atoms in Abplatin^(IV)^, making it possible to quantitatively study the intracellular uptake of Abplatin^(IV)^ by ICP-MS. The results showed that the Pt uptake by BEL-7404 cells gradually increased over time. Specifically, it was 1303 ng Pt/million cells at 7 h for Abplatin^(IV)^, while this was only 39 ng Pt/million cells and 948 ng Pt/million cells for cisplatin and CisPt(IV), which was 33.4 and 1.4 fold-increase in uptake for Abplatin^(IV)^ (Fig. 2C). Cisplatin could bind with DNA to form adducts, thereby affecting DNA replication and killing cancer cells [31, 32]. To investigate whether the increase Pt-DNA in Pt uptake of Abplatin^(IV)^ could result in more Pt-DNA adducts, DNA was extracted from BEL-7404 cells treated with various drugs, and the Pt-DNA adducts were tested by ICP-MS. The results showed that the Pt-DNA adducts of cells treated with Abplatin^(IV)^ was 3.22 and 1.15 times more than those treated with cisplatin and CisPt(IV) (Fig. 2D). The above results together showed that cancer cells treated by Abplatin^(IV)^ could promote the formation of Pt-DNA adducts, thereby resulting in more DNA damage and inhibiting cell proliferation[32].

We continued to evaluate the killing effect of Abplatin^(IV)^. The results showed that Abplatin^(IV)^ were much effective in killing cancer cells than cisplatin and CisPt(IV) on all cell lines tested (Fig. 2F, Additional file 1: Fig. S4, Table S1). Taking A2780 cell (human ovarian cancer cell) as an example, the IC_50_ of Abplatin^(IV)^, CisPt(IV), and cisplatin were 1.35 µM, 1.60 µM and 25.10 µM, respectively. For BEL-7404 cell, the IC_50_ of Abplatin^(IV)^ was much lower than that of CisPt(IV) and cisplatin. Even to cisplatin-resistant cells, like A2780DDP and BEL-7404DDP, Abplatin^(IV)^ still had the killing capability. Besides, we also compared the killing effect of Abplatin^(IV)^ with previously published drugs. The IC_50_ of Abplaitn^(IV)^ in A549 was smaller than that of the published Pt(IV) drugs (Additional file 1: Table S1) [9, 10, 18]. Furthermore, the apoptosis test showed that Abplatin^(IV)^ (10 μM) induced an apoptosis rate of 90.2% on BEL-7404 cells. However, at the same concentration, cisplatin and CisPt(IV) only induced an apoptosis rate of 11.1% and 38.9%, respectively (Additional file 1: Fig. S5).

To test whether Abplatin^(IV)^ could enter tumors and induce cell death, a 3D cell sphere was cultured to mimic the process. The calcein AM/PI (acetyl methoxy methyl ester/ propidium iodide) cell viability kit was used. Green fluorescence could be detected in living cells while red fluorescence could be detected in the dead cells. The results showed that the BEL-7404 cells in a 3D cell sphere after treated with Abplatin^(IV)^ showed strong red fluorescence(Fig. 2G), indicating the excellent cytotoxic of Abplatin^(IV)^.

### In vivo anticancer efficacy of Abplatin^(IV)^

**Fig. 3:**
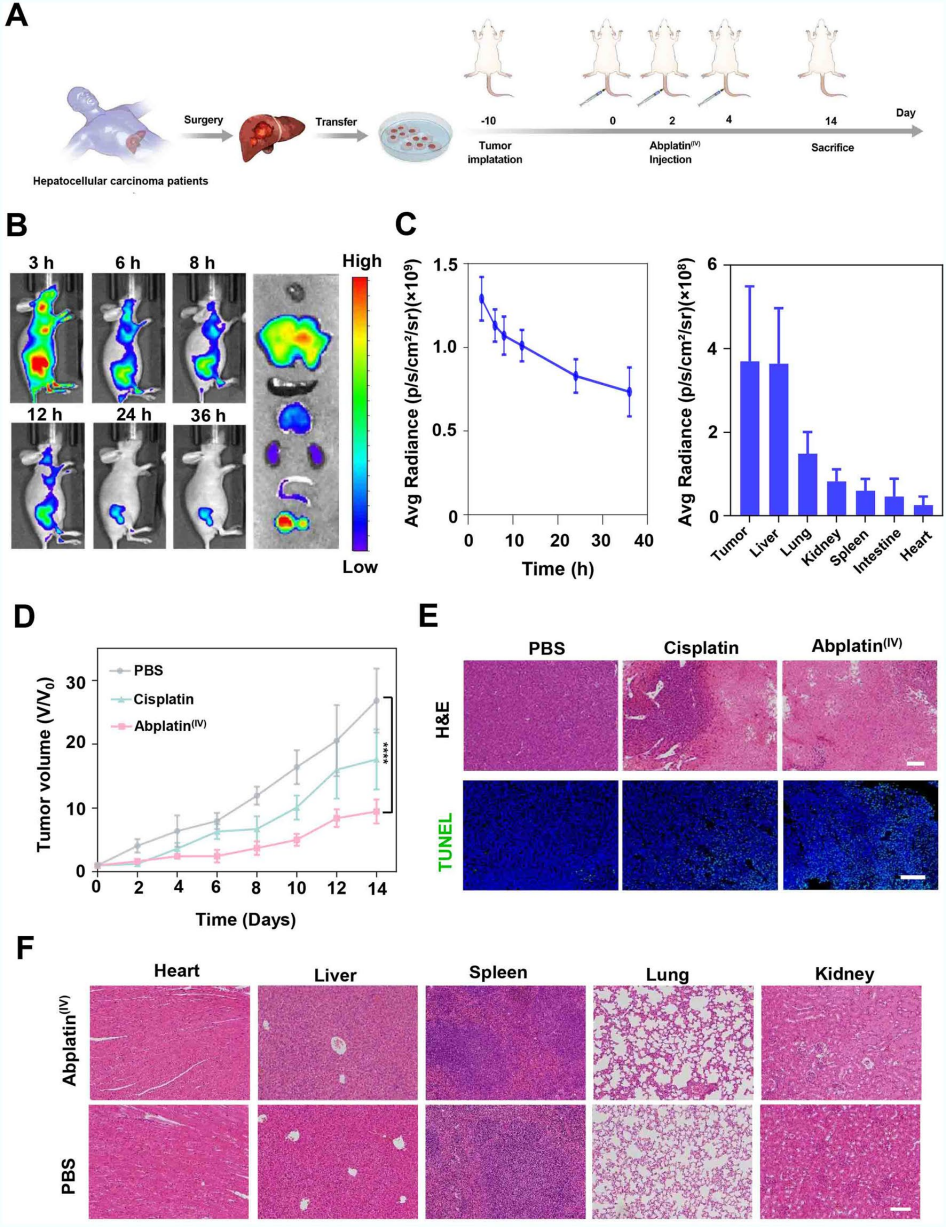
Abplatin^(IV)^ inhibited tumor growth on a PDX^HCC^ model. **A** Schematic illustration showing the establishment of a PDX^HCC^ model for in vivo studies. **B** In vivo biodistribution of Abplatin^(IV)^ labeled with Cy7.5 via in vivo imaging and ex vivo imaging of major organs at 36 h. **C** The corresponding semi-quantification of the fluorescence signal of Abplatin^(IV)^ over time and biodistribution of major organs. **D** Tumor growth inhibition curves of mice treated with PBS, cisplatin, and Abplatin^(IV)^. **E** H&E staining and TUNEL of representative tumor tissues. Scale bar for H&E images = 200 µm, scale bar for TUNEL images = 100 µm. **F** H&E staining of major organs of mice treated by PBS and Abplatin^(IV)^, scale bar = 100 µm

 We continued to evaluate the anti-tumor effect of Abplatin^(IV)^ on a PDX^HCC^ model. PDX model is a human xenotransplantation model established by directly transplanting liver tumor tissue from patients to immunodeficient mice (Fig. 3A). Such model retains the characteristics of patient tumors at the level of histopathology, molecular biology, and genetics, which is currently the preferable model for drug evaluation, and has been widely used to screen for anti-cancer drugs [15, 18].

We first carried out biodistribution study to investigate the tumor targeting of Abplatin^(IV)^. To track the nanoparticles through fluorescence signal, Abplatin^(IV)^ was labelled with Cy7.5, and i.v. injected into the tumor-bearing mice. As shown in Fig. 3B, Abplatin^(IV)^ were evenly distributed in the whole body at 3 h. Then, Abplatin^(IV)^ began to accumulate at the tumor site at 6 h, indicating their excellent tumor-targeting ability. After 36 h, the mice were sacrificed and all the major organs (i.e., heart, liver, spleen, lung and kidney) were collected. Ex vivo imaging showed that Abplatin^(IV)^ mainly accumulated in the liver and tumor regions (Fig. 3C).

For tumor inhibition study, Abplatin^(IV)^ was *i.v.* injected into the mice and the tumor volume was measured every 2 days. The tumor inhibition curve indicated that Abplatin^(IV)^ had the most obvious tumor inhibition effect, followed by cisplatin (Fig. 3D). The tumor inhibition rate was further calculated to be 34.3% and 64.8% for cisplatin and Abplatin^(IV)^ respectively. Subsequent hematoxylin and eosin (H&E) staining on the tumor tissues showed the cells in the tumor tissue in mice treated with PBS presented a rich and full state, while the cells in the tumor tissues in mice treated with cisplatin and Abplatin^(IV)^ presented a nuclear shrinkage state. Further terminal deoxynucleotidyl transferase dUTP nick end labeling (TUNEL) staining also showed that the tumor tissues treated with Abplatin^(IV)^ had the most severe apoptosis (Fig. 3E).

The toxic side effects of Abplatin^(IV)^ were further investigated. Fristly, the blood physiological parameters of the mice were monitored. The results showed that the blood physiological and biochemical indexes were normal (Additional file 1: Fig. S6). Subsequently H&E staining demonstrated that the heart, liver, spleen, lung and kidney did not show morphological damage (Fig. 3F). Overall, excellent biosecurity of Abplatin^(IV)^ has been demonstrated with the above studies.

### RNA-seq analysis in BEL-7404 cells treated with Abplatin^(IV)^

**Fig. 4:**
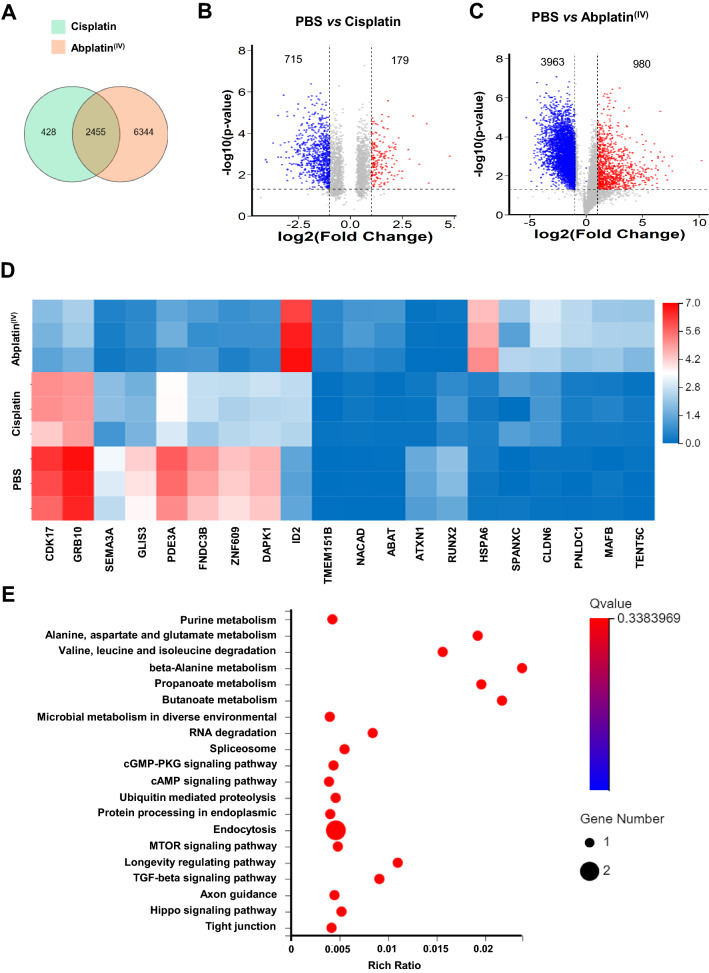
Transcriptomic analysis of BEL-7404 cells treated with cisplatin and Abplatin^(IV)^ by RNA-seq. **A** A Venn diagram revealed the number of genes transcribed in each treatment group. **B**, **C** Volcano plots displayed the differentially expressed genes. **D** A heat map showed the significant differential transcription genes. **E** Kyoto encyclopedia of genes and genomes (KEGG) pathway enrichment analysis based on differential genes

The molecular mechanism of cisplatin had been extensively studied. It was found that p53 activated pro-apoptotic genes and resulted in apoptosis when cells were treated by cisplatin [33]. In addition, it was proposed that caspase-6 and -7 genes were the transcriptional targets of p53 in cisplatin injury [34]. However, although there were numerous reports on Pt(IV) drugs and their DDS, the molecular mechanism of them is still controversial. To understand this, RNA-seq analysis was performed on BEL-7404 cells treated with PBS, cisplatin, and Abplatin^(IV)^. We found that there are 9227 genes in the transcription detected. Among those genes, 428 genes were specifically transcribed in the cells treated with cisplatin, while 6344 genes were exclusively transcribed in the cells treated with Abplatin^(IV)^ (Fig. 4A). Compared with the cells treated with PBS, 179 genes were up-regulated (red dots) and 715 genes were down-regulated (blue dots) in the cells treated with cisplatin (Fig. 4B). However, there were 980 genes up-regulated and 3963 genes down-regulated in the cells treated with Abplatin^(IV)^ (Fig. 4C). Obviously, Abplatin^(IV)^ treatment resulted in more genetic disorders than cisplatin. Thereafter, we only selected 20 genes with significant differences for heat map analysis. The results showed that genes such as HSPA6, ABAT, PNLDC1, TENT5C, CLDN6, TMEM151B, ID2, NACAD, SPANXC, and MAFB were significantly up-regulated, while SEMA3A, FNDC3B, ATXN1, ZNF609, DAPK1, GLIS3, CDK17, PDE3A, GRB10, and RUNX2 were significantly down-regulated (Fig. 4D, Additional file 1: Table S2). Moreover, it was reported that the overexpression of ABAT gene could significantly reduce cell proliferation and migration, and impair the production of lactic acid [35]. The increased expression of ABAT in the Abplatin^(IV)^-treated cells might be the one of reasons for its cell killing effect. Claudin 6 (CLDN6), a member of the Claudin (CLDN) family of tight junction proteins, was reported to be able to inhibit the proliferation and induce apoptosis of cancer cells [36]. We found here CLDN6 was also highly expressed in cells treated with Abplatin^(IV)^. Subsequent Kyoto Encyclopedia of Genes and Genomes (KEGG) enrichment analysis further demonstrated that purine metabolism, alanine, aspartate, and glutamate metabolism, etc*.*, were mainly affected in BEL-7404 cells treated with Abplatin^(IV)^ (Fig. 4E).

### Lipidomics and metabolomics analysis in BEL-7404 cells treated with Abplatin^(IV)^

**Fig. 5:**
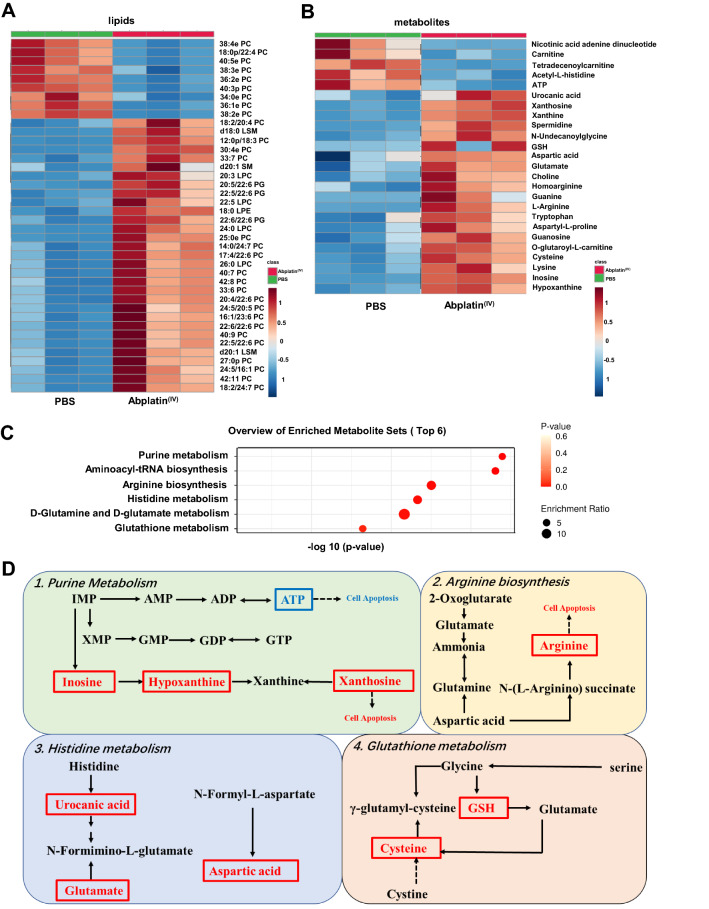
Lipidomic and metabolomic study on BEL-7404 cells treated with Abplatin^(IV)^. **A** Differential lipids between PBS and Abplatin^(IV)^ treated cells. **B** Differential metabolites between PBS and Abplatin^(IV)^-treated cells. **C** Overview of enriched metabolite sets in the cells treated with Abplatin^(IV)^ and PBS. **D** Abplatin^(IV)^ disturbed the purine metabolism, arginine biosynthesis, histidine metabolism, and glutathione metabolism in BEL-7404 cells. Up-regulated and down-regulated metabolites were represented in red and blue, respectively

To further illustrate the molecular mechanism of Abplatin^(IV)^, lipidomic and metabolomic studies were performed. The representative total ion chromatograms (TICs) of BEL-7404 lipids and metabolites were shown in Additional file 1: Figs. S7 and S8. Further, the partial least-squares discriminant analysis (PLS-DA) score plots in positive and negative ion detection mode indicated that the cells treated with PBS, cisplatin, and Abplatin^(IV)^ were different and could be distinguished (Additional file 1: Figs. S9 and S10). There were 40 differential lipids between Abplatin^(IV)^ and PBS treatment groups (Fig. 5A), while there were 31 differential lipids between cisplatin and PBS treatment groups (Additional file 1: Fig. S11A). Specifically, in the Abplatin^(IV)^ treated group, twenty phosphatidylcholine (PCs), three phosphatidylglycerols (PGs), four lysophosphatidylcholines (LPCs), one lysophosphatidylethanolamine (LPE), one sphingomyelin (SM) and two lysosphingomyelins (LSMs) were up-regulated compared with the PBS treatment group. Moreover, we found here there were nine PCs were down-regulated in the Abplatin^(IV)^ treatment group compared with the PBS treatment group (Additional file 1: Table S3). Notably, the main differential lipids were glycerophospholipids (LPC, LPE, PC, and PG) and a very small amount of sphingolipids (SM, LSM) in the cells treated with Abplatin^(IV)^ compared with PBS. For glycerides and sterol lipids, there were no significant changes. As the glycerophospholipids and sphingolipids were the main components of cell membrane, the disorder induced by Abplatin^(IV)^ revealed by lipodomics might indicate the changes of BEL-7404 cell membrane.

Metabolomics analysis on BEL-7404 treated with Abplatin^(IV)^ and cisplatin was then performed. The results showed that there were 25 differential metabolites between Abplatin^(IV)^ and PBS treatment groups (Fig. 5B), while there were 30 differential metabolites between cisplatin and PBS treatment groups (Additional file 1: Fig. S11B). We found that among these differential metabolites, there were 14 common differential metabolites, such as ATP, nicotinic acid adenine dinucleotide, glutamate, hypoxanthine, etc*.* (Additional file 1: Table S4). Moreover, these common differential metabolites had the similar trend in change. Specifically, ATP and nicotinic acid adenine dinucleotide were down-regulated and others were up-regulated in the cells treated with Abplatin^(IV)^ and cisplatin. Since Pt(IV) drugs can be reduced to Pt(II) to exert anticancer efficacy, part of metabolites showed the same change trend in the two treatment groups.

 There were 25 metabolites significantly changed in the cells treated with Abplatin^(IV)^ (Additional file 1: Table S5). Further metabolic pathways analysis indicated that purine metabolism, arginine biosynthesis, histidine metabolism, and glutathione metabolism were mainly affected (Fig. 5C and D). Compared with cisplatin, Abplatin^(IV)^ more significantly disturbed the purine metabolism pathway (Additional file 1: Fig. S12). In purine metabolism pathway, ATP was down-regulated and xanthosine and hypoxanthine were up-regulated. As purine was the basic component of nucleotides in cell proliferation, the impaired purine metabolism pathway might be related to cancer progression [37]. ATP is the energy source of living cells [38], and the decreased ATP in the cells treated with Abplatin^(IV)^ indicated there was insufficient cell energy, possibly resulting in cell apoptosis. It was reported that xanthosine could inhibit cancer cell proliferation and hypoxanthine could induce apoptosis by regulating the expression of apoptosis-related proteins [39, 40]. Therefore, the increase of hypoxanthine and xanthosine in Abplatin^(IV)^ treated cells might also be a cause for cell death. Moreover, we found here in arginine biosynthesis pathway, the aspartic acid and arginine in Abplatin^(IV)^ treated cells were significantly up-regulated and the carnitine content were down-regulated in the cells treated with Abplatin^(IV)^ compared with PBS. It was reported that the increase of arginine could also result in cell apoptosis and carnitine could improve energy status, reduce oxidative stress, and prevent subsequent cell death [41, 42]. Therefore, the results further indicated the increase of arginine and decrease of carnitine might result in cell apoptosis. Finally, homoarginine was found to be up-regulated in the cells treated with Abplatin^(IV)^ compared with PBS group, indicating that the increase of homoarginine could inhibit cell proliferation and caused cell death[40].

## Conclusion

Due to the importance of Pt(II)-based drugs in cancer therapy, Pt(IV) drugs and their DDS are very promising. Although the molecular mechanism of Pt(II)-based drugs such as cisplatin has been widely reported in the literature, there is very limited report on the molecular mechanism of Pt(IV)-based drugs, especially from the perspective of multi-omics. Therefore, in this paper, CisPt(IV) with two hydrophobic aliphatic chains in the axial position was chosen. Subsequently, the most common drug carrier HSA was adopted to encapsulate it to form Abplatin^(IV)^. Thereafter, we explored the anticancer effect of Abplatin^(IV)^ on cancer cells, 3D cell sphere and on a PDX^HCC^ mice model. In vitro, the IC_50_ of Abplatin^(IV)^ was 1.35 µM on A2780 cells, much lower than that of cisplatin at 25.10 µM. Abplatin^(IV)^ exhibit excellent killing capability even on the cisplatin-resistant cells such as A2780DDP and BEL-7404DDP cells. Moreover, our study showed that Abplatin^(IV)^ had better anticancer activity on 3D cell spheres, with enhanced Pt-DNA adducts formation, thereby resulting in greater DNA damage and inhibiting cell proliferation. In vivo, we established a PDX^HCC^ model, Abplatin^(IV)^ showed better tumor-targeting performance and tumor inhibition ability than cisplatin. Finally, a multi-omics strategy, including transcriptomics, metabolomics, and lipidomics, was carried out on BEL-7404 cells to elucidate the molecular mechanism of Abplatin^(IV)^. We found here that Abplatin^(IV)^ might induce the change of BEL-7404 cell membrane, hence resulting in the disorder of glycerophospholipids and sphingolipids. More importantly, many genes were affected, and the significant up-regulation of ABAT and CLDN6 genes might inhibit the cancer cells proliferation and induce cell apoptosis. Meanwhile, the transcriptomics and metabolomics study showed that Abplatin^(IV)^ significantly disturbed the purine metabolism pathway. We further found that in purine metabolism pathway, ATP was down-regulated, and xanthosine and hypoxanthine were up-regulated. In addition, arginine was increased, and carnitine was decreased after Abplatin^(IV)^ treatment, which might result in cell apoptosis as well. This research highlighted the development of Abplatin^(IV)^ and the related multi-omics analysis for the study of molecular mechanism of action, which might be helpful for the clinical translational of Pt(IV) drugs and their DDS in the future.

## Methods

### Materials and reagents

*N*-Octyl isocyanate and hydrogen peroxide (H_2_O_2_), dimethylthiazol-2-yl)-2,5-diphenyltetrazolium bromide (MTT) were purchased from Aladdin (Shanghai, China). Cisplatin (purity 99%) was bought from Kunming Institute of Precious Metals (Yunnan, China). Human serum albumin (HSA) was purchased from Shang Hai Yuan Ye Bio-Technology Co., Ltd (Shanghai, China). 2-(4-Amidinophenyl)-1H-indole-6-carboxamidine (DAPI), Alexa Fluor 488 (Alex 488), Cy5.5, and Cy7.5 were purchased from Sigma-Aldrich (Shanghai, China). Calcein AM/PI (acetyl methoxy methyl ester/propidium iodide) cell viability kit, DNA extraction kit and Annexin V-FITC apoptosis detection kit was purchased from Beyotime Institute of Biotechnology (Jiangsu, China). Terminal deoxynucleotidyl transferase dUTP nick end labeling (TUNEL) kit were purchased from Roche (Basel, Switzerland). RPMI-1640 medium, DMEM, 0.25% trypsin–EDTA, fetal bovine serum (FBS), and penicillin/streptomycin (P/S) were purchased from Gibco (NY, USA). Cell culture vessels were purchased from Corning (NY, USA).

### In vivo biodistribution imaging

Abplatin^(IV)^ labelled with Cy7.5 was i.v. injected into the mice. At designed time points post injection (3 h, 6 h, 8 h, 12 h, 24 h, and 36 h), the mice were imaged by an in vivo imaging system (IVIS) (PerkinElmer, E_x_/E_m_ = 780 nm/810 nm, Waltham, USA) to detect the Abplatin^(IV)^ accumulation. After 36 h of injection, mice were sacrificed and the main organs including heart, liver, lung, kidney, spleen and tumors were isolated and used for ex vivo imaging.

### Therapeutic effect of Abplatin^(IV)^ on a hepatocellular carcinoma patient-derived tumor xenograft (PDX^HCC^) model

PDX^HCC^ model was established as previously described [43, 44]. When the subcutaneous tumors reached approximately 100 mm^3^, the mice were randomly divided into three groups with 5 mice in each group. The drugs were i.v*.* injected in the day 0, 2, and 4. Treatment groups were: (1) PBS, (2) cisplatin (3.5 mg Pt/kg), (3) Abplatin^(IV)^ (3.5 mg Pt/kg). The tumor volume were recorded every other day. The tumor volume was calculated following the equation below: tumor volume = 1/2 × LW^2^, where “L” refers to the long diameter of the tumor and “W” means the short diameter of the tumor. The relative tumor volume was set as the V_t_/V_0_. “V_t_” refers to the mean tumor volume at a desirable day when tumor volumes were collected. “V_0_” refers to total mean value of the initial tumor volume.

### In vivo toxicity evaluation

Healthy female Kunming (KM) mice were randomly assigned to 2 groups (n = 3) with injection of PBS and Abplatin^(IV)^ at a dose of 3.5 mg Pt/kg body weight via the tail vein. The mice were weighted every other day and sacrificed 10 days post the first administration. In order to assess the potential toxicity of Abplatin^(IV)^, histology analysis was performed on major organs including heart, spleen, liver, kidney, and lung. Tissues from these organs were collected, fixed with 4% paraformaldehyde solution, and sliced for H&E staining assay. Meanwhile, blood biochemistry assays were conducted to quantitatively evaluate the potential kidney, heart and liver function as follows: blood urea nitrogen (BUN) (indicators for kidney function), creatinine (CRE) (indicators for kidney function), aminotransferase (AST) (indicators for hepatic function), creatine kinase (CK) (indicators for heart function), aminotransferase (ALT) (indicators for hepatic function), and creatine kinase MB (CKMB) (indicators for heart function).

### Statistical analysis

Student’s *t* test was used to determine the statistical difference between groups. Differences were considered statistically significant at a level of *p < 0.05; **p < 0.01; ***p < 0.001; ****p < 0.0001.

## Supplementary Information

**Additional file 1: Figure S1.** Synthesis route and chemical structures of CisPt(IV). **Figure S2.** Characterization of CisPt(IV) by ESI-MS (positive mode). **Figure S3.** X-ray photoelectron spectroscopy (XPS) analysis of sulfur element in CisPt(IV) (A) and Abplatin^(IV)^ (B). **Figure S4.** In vitro anticancer activity of cisplatin, CisPt(IV), and Abplatin^(IV)^. **Figure S5.** Total percentages of BEL-7404 cells that underwent apoptosis as determined by flow cytometery after treatment with various Pt-containing drugs. **Figure S6.** Blood physiological and biochemical indexes of mice treated by PBS, cisplatin, and Abplatin^(IV)^. **Figure S7.** The representative total ion chromatograms (TIC) of lipids extracted from the BEL-7404 cells in the positive ion mode (A) and negative ion mode (B). **Figure S8.** The representative total ion chromatograms (TIC) of metabolites extracted from the BEL-7404 cells in the positive ion mode (A) and negative ion mode (B). **Figure S9.** PLS-DA plots for discriminating the PBS, cisplatin, and Abplatin^(IV)^ group at the lipid level in BEL-7404 cells obtained by UPLC-ESI(+)-Orbitrap-MS (A) and UPLC-ESI(-)-Orbitrap-MS (B). **Figure S10.** PLS-DA plots for discriminating the PBS, cisplatin, and Abplatin^(IV)^ group at the metabolite level in BEL-7404 cells obtained by UPLC-ESI(+)-Qrbitrap-MS (A) and UPLC-ESI(-)-Orbitrap-MS (B). **Figure S11.** (A) Differential lipids between PBS and cisplatin-treated cells. (B) Differential metabolites between PBS and cisplatin-treated cells. **Figure S12.** (A) Metabolite sets enrichment overview of differential cells affected by cisplatin compared with PBS. (B) Metabolite sets enrichment overview of differential cells affected by Abplatin^(IV)^ compared with PBS. **Table S1.** IC50 values of various Pt-containing drugs on various cell lines. **Table S2.** The significant differential genes in Abplatin^(IV)^ treated group (s) compared with the PBS group (c). **Table S3.** The differential lipids in Abplatin^(IV)^ treated group (s) compared with the PBS group (c). **Table S4.** The common differential metabolites in Abplatin^(IV)^ treated group (s2), cisplatin treated group (s1) compared with the PBS group (c). **Table S5.** The mainly differential metabolites in Abplatin^(IV)^ treated group (s) compared with the PBS group (c).

## Data Availability

Not applicable.

## Abbreviations

Pt(II) drugs: Platinum(II) drugs
Pt(IV) drugs: Platinum(IV) drugs
HSA: Human serum albumin
CisPt(IV): Hydrophobic Pt(IV) prodrug
AbPlatin^(IV)^: CisPt(IV) encapsulated by HSA
