# Supplementary material for: Abplatin(IV) inhibited tumor growth on a patient derived cancer model of hepatocellular carcinoma and its comparative multi-omics study with cisplatin
Source: J Nanobiotechnology. 2022 Jun 3;20:258. doi: 10.1186/s12951-022-01465-y (PMC9164404; doi:10.1186/s12951-022-01465-y)
Supplement: Supplementary file 1 — Additional file 1: Figure S1. Synthesis route and chemical structures of CisPt(IV). Figure S2. Characterization of CisPt(IV) by ESI-MS (positive mode). Figure S3. X-ray photoelectron spectroscopy (XPS) analysis of sulfur element in CisPt(IV) (A) and Abplatin(IV) (B). Figure S4. In vitro anticancer activity of cisplatin, CisPt(IV), and Abplatin(IV). Figure S5. Total percentages of BEL-7404 cells that underwent apoptosis as determined by flow cytometery after treatment with various Pt-containing drugs. Figure S6. Blood physiological and biochemical indexes of mice treated by PBS, cisplatin, and Abplatin(IV). Figure S7. The representative total ion chromatograms (TIC) of lipids extracted from the BEL-7404 cells in the positive ion mode (A) and negative ion mode (B). Figure S8. The representative total ion chromatograms (TIC) of metabolites extracted from the BEL-7404 cells in the positive ion mode (A) and negative ion mode (B). Figure S9. PLS-DA plots for discriminating the PBS, cisplatin, and Abplatin(IV) group at the lipid level in BEL-7404 cells obtained by UPLC-ESI(+)-Orbitrap-MS (A) and UPLC-ESI(-)-Orbitrap-MS (B). Figure S10. PLS-DA plots for discriminating the PBS, cisplatin, and Abplatin(IV) group at the metabolite level in BEL-7404 cells obtained by UPLC-ESI(+)-Qrbitrap-MS (A) and UPLC-ESI(-)-Orbitrap-MS (B). Figure S11. (A) Differential lipids between PBS and cisplatin-treated cells. (B) Differential metabolites between PBS and cisplatin-treated cells. Figure S12. (A) Metabolite sets enrichment overview of differential cells affected by cisplatin compared with PBS. (B) Metabolite sets enrichment overview of differential cells affected by Abplatin(IV) compared with PBS. Table S1. IC50 values of various Pt-containing drugs on various cell lines. Table S2. The significant differential genes in Abplatin(IV) treated group (s) compared with the PBS group (c). Table S3. The differential lipids in Abplatin(IV) treated group (s) compared with the PBS group (c). Table [file 12951_2022_1465_MOESM1_ESM.docx]

**Additional Information**

**Abplatin^(IV)^ Inhibited Tumor Growth on a** **Patient Derived Cancer Model of Hepatocellular Carcinoma and its Comparative Multi-omics Study with Cisplatin**

Xing Li^1,5^, Lingpu Zhang^1,2^, Tuo Li^1,3^, Shumu Li^1^, Wenjing Wu^1,5^, Lingyu Zhao^1,5^, Peng Xie^4^, Jinqi Yang^1,5^, Peipei Li^5^, Yangyang Zhang^1^, Haihua Xiao^5^, Yingjie Yu^2,*^ and Zhenwen Zhao^1,5,*^

^1^ Beijing National Laboratory for Molecular Sciences, CAS Research/Education Center for Excellence in Molecular Sciences, Institute of Chemistry Chinese Academy of Sciences, Beijing Mass Spectrum Center, Beijing, 100190, China

^2^ College of Life Science and Technology; State Key Laboratory of Organic-Inorganic Composites; Beijing University of Chemical Technology, Beijing 100029, China

^3^ Department of Nuclear Medicine, Peking Union Medical College Hospital, Chinese Academy of Medical Science & Peking Union Medical College, Beijing, 100730, China

^4^ Department of orthopedics, The Second Xiangya Hospital, Central South University, Changsha, Hunan 410011, China

^5^ Graduate School, University of Chinese Academy of Sciences, Beijing, 100049, China

^*^Corresponding author.

Email addresses: yuyingjie@mail.buct.edu.cn (Y. Yu), [zhenwenzhao@iccas.ac.cn](mailto:zhenwenzhao@iccas.ac.cn) (Z. Zhao).

**General Measurements**

^1^H NMR spectrum was measured by a 400 MHz NMR spectrometer (Bruker, Germany). The morphology of Abplatin^(IV)^ were measured by transmission electron microscopy (TEM) (JEM-2100F, JEOL, Japan). Particle size was conducted on a Malvern Zetasizer (Nano ZS, UK). Composition of the nanoparticles were conducted using a scanning transmission electron microscopy (STEM) equipped with an energy dispersive spectroscopy detector (JEOL 2100F, JEOL, Japan). Localiztion of nanoparticles were peformed using a confocal laser scanning microscopy (ZEISS LSM880, Germany). Flow cytometry was conducted by Cytomics FC500 Flow Cytometry (Beckman Coulter Ltd., USA). Quantitative analysis of platinum contents was measured by Inductively coupled plasma mass spectrometer (ICP-MS) (Agilent technologies 7700 series, USA). The lipidome and metabolome analysis was performed using UPLC-ESI-Qrbitrap-MS (Thermo Fisher Scientifc, USA). The mice imaging was conducted by an *in* *vivo* Imaging System (IVIS) (Waltham, USA.).

**Cell lines and animals**

A2780 (cisplatin sensitive ovarian cancer cell line), A2780DDP (cisplatin-resistant ovarian cancer cell line), BEL-7404(cisplatin sensitive liver cancer cell line), and BEL-7404DDP (cisplatin-resistant liver cancer cell line) were kindly supplied by the National Center for Nanoscience and Technology in China.

Female BALB/c nude mice and female Kunming (KM) mice (6~8 weeks old) were purchased from Beijing Vital Lihua Company. All animal experiments were conducted under guidelines evaluated and approved by Peking University Institutional Animal Care and Use Committee (LA2021316)

**Synthesis of** **CisPt(IV) and Abplatin^(IV)^**

CisPt(IV) was prepared according to our previous work [1]. Abplatin^(IV)^ was prepared as follows: 3 mg/mL human serum albumin (HSA) aqueous solution and 10 mg/mL of CisPt(IV) DMSO solution were firstly prepared. 50 µL of CisPt(IV) solution was added dropwise into a mixture of HSA solution and stirred for 10 mins. Then the solution was transferred into ultrafiltration centrifugal tube, and centrifuged at 3000 rpm/min for 5 min. After centrifugation, the supernatant was placed in a dialysis bag (MWCO: 3500 Da) and then dialysis to remove DMSO.

**Drug Release of Abplatin^(IV)^.**

Abplatin^(IV)^ aqueous solution (5 mL, 100 µM) was transferred to a pre-swelled dialysis bag (MWCO: 3500 Da) and immersed in three different mediums (PBS (1X, pH 7.4), acetate buffer solution (pH 5.0), and sodium ascorbate solution (5 mM NaVc), 100 mL) in a 37°C oscillating incubator. At each time point, 1 mL of sample solution was taken from the medium for measurement of Pt drug released by ICP-MS, and meanwhile 1 mL of fresh PBS solution was added into the medium to keep the total volume unchanged. The amount of Pt released in the sample can be calculated by comparison with the total amount of Pt in the dialysis bag before dialysis.

***In Vitro* Cytotoxicity**

Human hepatocellular carcinoma (BEL-7404 and BEL-7404DDP) and human ovarian cancer (A2780 and A2780DDP) were seeded in 96-well plates (8×10^3^ cells/100 μL) and incubated for 12 h. Then the cells were treated with PBS, cisplatin, CisPt(IV), and Abplatin^(IV)^ at various Pt concentrations ranging from 0.005 µM to 40 µM for 48 h. MTT reagent (10 µL of a 5 mg/mL solution in PBS buffer) was added into well for 4 h. Thereafter, 10% SDS (100 µL/well) was added and incubated for 12 h. Measurements of absorbance were subsequently performed with a Microplate reader (SpectraMax, USA) at 570 nm (peak absorbance) and subtracted at 650 nm (background absorbance).

**The uptake and apoptosis test on 3D tumor spheres**

1% agarose gel solution (50 μL) was added into each well of 96-well plate. 1600 cells (200 μL) were sequentially seeded. At Day 7, the cell spheres were basically formed, and then treated with cisplatin, CisPt(IV), and Abplatin^(IV)^ at the concentration of 10 μM for 24 h before stained by Calcein AM/PI cell viability kit. Cells were then observed by a fluorescence microscope (ZEISS, Germany).

**Cellular uptake of Abplatin^(IV)^ by confocal laser scanning microscopy (CLSM) and flow cytometry.**

To track Abplatin^(IV)^ in the cells, Abplatin^(IV)^ was labelled with fluorescent dye Cy5.5 (termed as Abplatin^(IV)^@Cy5.5) according to our previous work [2]. BEL-7404 cells at a density of 5×10^4^ per well were seeded in a 24-well plate, and incubated at 37°C overnight. The cells were exposed to Abplatin^(IV)^@Cy5.5 with a final concentration of 100 ng/mL of Cy5.5 for 1 h, 4 h, and 7 h. After that, cells were rinsed with cold PBS and fixed with paraformaldehyde. To better evaluate the cell colocalization, the nuclei were stained with DAPI (blue) and cytoskeleton were strained with Alexa Fluor 488 (green), respectively.

The intracellular uptake studies of Abplatin^(IV)^ were also performed using flow cytometry. BEL-7404 cells were seeded in 12-well plates with a density of 3×10^5^ cells per well. After incubation overnight, cells were exposed to the above synthesized Abplatin^(IV)^@Cy5.5 for another 1 h, 4 h, and 7 h at 37 °C. Next, cells were rinsed with PBS and harvested by trypsin, and fluorescence intensity was detected by flow cytometry.

**Platinum Uptake in the Cell**

BEL-7404 cells (1×10^6^) were seeded in 6-well plates and cultured. After incubation overnight, the cells were treated with cisplatin, CisPt(IV) or Abplatin^(IV)^ (10 μM) in the culture medium at 37 °C for 1 h, 4 h, and 7 h, respectively. The cells were then rinsed three times with PBS and lysed with lysis buffer. Thereafter, Pt content was measured by ICP-MS.

**Pt-DNA adduct assay**

BEL-7404 cells were seeded in 6-well plates at a density of 1×10^6^ per well and incubated at 37 °C for 12 h. Cisplatin, CisPt(IV) or Abplatin^(IV)^ were added into the wells at a final concentration of 40 µM Pt. After incubation for 4 h at 37 °C, cells were rinsed three times with cold PBS, and DNA was extracted by using a DNA extraction kit. DNA yields were evaluated by NanoDrop 2000 spectrophotometer (Thermo Fisher Scientific, USA) and Pt contents were determined *via* ICP-MS.

**Cellular Apoptosis Assays**

BEL-7404 cells were seeded in 12-well plates at a density of 3×10^4^/mL and incubated with various treatments at a fix Pt concentration (10 μM). After 24 h, apoptotic cells were detected by flow cytometery, using the Annexin V-FITC Aopoptosis Detection Kit.

**RNA-seq Analysis.**

BEL-7404 cells (2 × 10^6^) were seeded in 6-well plates and cultured for 12 h. Then the cells were treated with PBS, cisplatin (5 μM Pt) or Abplatin^(IV)^ (5 μM Pt) for 12 h. BGISEQ-500 was used for RNA-seq. RSEM was utilized to quantify the transcription levels. Differentially expressed genes (DEGs) were identified when fold changes were greater than or equal to 2 and the p-values were less than or equal to 0.05. GraphPad Prism software and MetaboAnalyst website (https://www.metaboanalyst.ca/) to generate volcano graphs and the heatmap, respectively. Significant enrichments were identified when the Q values were less than or equal to 0.05.

**Metabolomics and lipidomics Analysis.**

PBS, cisplatin (5 μM Pt) or Abplatin^(IV)^ (5 μM Pt)-treated BEL-7404 cells (6 × 10^6^) for 12 h were collected. 100 µL of water, 180 µL of methanol, and 120 µL chloroform were added for extraction of metabolome and lipidome. The mixed solution were vortexed for 15 s and standed for 15 s. Then 150 µL water were added again to promote phase separation. The solution were vortexed for 1 min and standed for 5 min. Thereafter, the solution was centrifuged (15 min, 10,000 g). 350 µL of upper layer solution was dried by vacuum centrifugal drying, and then re-dissolve with 40 µL water (containing 20 mM ammonium acetate) for metabolome analysis. At the same time, 50 µL of lower layer solution was obtained for lipidome analysis.

Ultra-performance liquid chromatography (UPLC, Ultimate 3000, Thermo Fisher Scientific, San Jose, CA, USA)-electrospray ionization -MS (Orbitrap Fusion Lumos, Thermo Fisher Scientific, San Jose, CA, USA) was used for metabolomics and lipidomics analysis. for metabolomics analysis, BEH Amide column (1.7 μm, 2.1 × 100 mm, Waters) was chosen for the separation of the metabolism. The column was maintained at 25℃. The mobile phase A was H_2_O containing 10 mM of ammonium acetate, and the mobile phase B was acetonitrile containing 0.1% formic acid. The UPLC separations were 30 min/sample using the following scheme: (1) 0 min, 95% B; (2) 20 min, 70% B; (3) 21 min, 50% B; (4) 24 min, 50% B; (5) 25 min, 95% B; (6) 30 min, 95% B. All the changes were linear, and the ﬂow rate was set to 300 μL/min. The method of lipidomics was referred to our previous work [3]. The raw data of LC-ESI-MS/MS were processed by using Compound Discoverer and LipidSearch software for automated signal extraction, identification and relative quantification of the metabolic small molecules and lipid compounds. At the same time, the data were imported into SIMCA-P software (version 14.1, Umetrics AB, Umea, Sweden) for multivariate pattern recognition analysis.

**Figure S1**. Synthesis route and chemical structures of CisPt(IV).

**Figure S2**. Characterization of CisPt(IV) by ESI-MS (positive mode). (A) The full mass spectrum of CisPt(IV). (B) Experimental isotopic pattern of CisPt(IV).

**Figure S3**. X-ray photoelectron spectroscopy (XPS) analysis of sulfur element in CisPt(IV) (A) and Abplatin^(IV)^ (B).

**Figure S4**. *In vitro* anticancer activity of cisplatin, CisPt(IV), and Abplatin^(IV)^.

**Figure S5.** Total percentages of BEL-7404 cells that underwent apoptosist as determined by flow cytometery after treatement with various Pt-containg drugs.

**
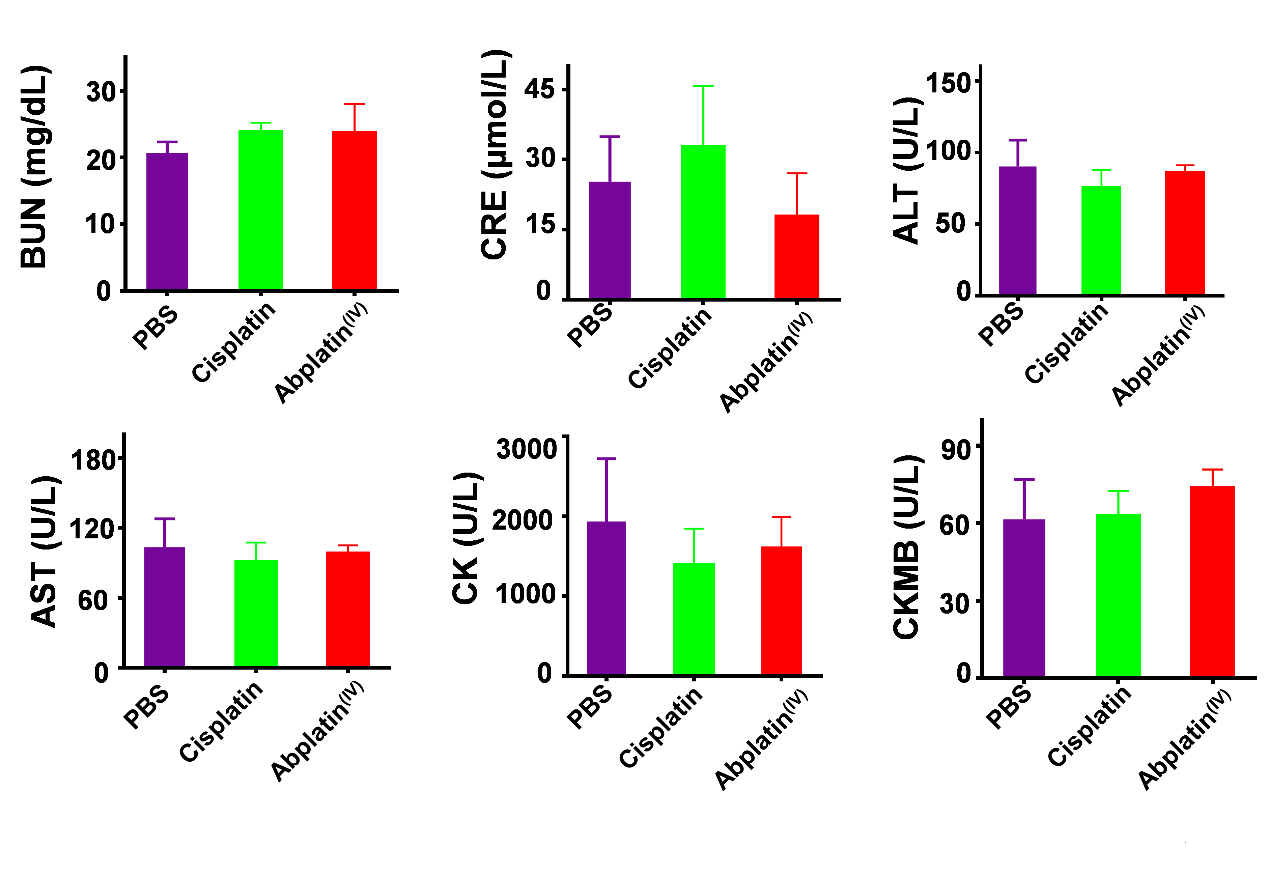
**

**Figure S6**. Blood physiological and biochemical indexes of mice treated by PBS, cisplatin, and Abplatin^(IV)^.

**Figure S7**. The representative total ion chromatograms (TIC) of lipids extracted from the BEL-7404 cells in the positive ion mode (A) and negative ion mode (B).

**Figure S8**. The representative total ion chromatograms (TIC) of metabolites extracted from the BEL-7404 cells in the positive ion mode (A) and negative ion mode (B).

**Figure S9**. PLS-DA plots for discriminating the PBS, cisplatin, and Abplatin^(IV)^ group at the lipid level in BEL-7404 cells obtained by UPLC-ESI(+)-Orbitrap-MS (A) and UPLC-ESI(-)-Orbitrap-MS (B).

**Figure S10**. PLS-DA plots for discriminating the PBS, cisplatin, and Abplatin^(IV)^ group at the metabolite level in BEL-7404 cells obtained by UPLC-ESI(+)-Qrbitrap-MS (A) and UPLC-ESI(-)-Orbitrap-MS (B).

**Figure S11**. (A) Differential lipids between PBS and cisplatin-treated cells. (B) Differential metabolites between PBS and cisplatin-treated cells.

**Figure S12**. (A) Metabolite sets enrichment overview of differential cells affected by cisplatin compared with PBS. (B) Metabolite sets enrichment overview of differential cells affected by Abplatin^(IV)^ compared with PBS.

**Table S1.** IC_50_ values of various Pt-containing drugs on various cell lines.

| Cell lines | IC_50_ (µM) | | | | | |
| --- | --- | --- | --- | --- | --- | --- |
|  | 24 h | | | | 48 h | |
|  | A2780 | A2780DDP | BEL-7404 | BEL-7404DDP | A549 | 7404 |
| Cisplatin | 25.10 | >40 | >>40 | >>40 | 4.91 | 10.43 |
| CisPt(IV) | 1.60 | 2.83 | >>40 | 12.9 | 3.06 | 3.45 |
| Abplatin^(IV)^ | 1.35 | 2.18 | 23.70 | 8.80 | 0.53 | 0.85 |
| NP2[4] | - | - | - | - | 1.54 | - |
| Compound A4[5] | - | - | - | - | 11.71 | - |
| Comound 3c[6] | - | - | - | - | 10.29 | - |

**Table S2**. The significant differential genes in Abplatin^(IV)^ treated group (s) compared with the PBS group (c).

FC, fold change; Dark grey: FC≥2; Light grey: FC≤0.5.

|  | **Aberrant gene ID** | **Aberrant gene symbol** | **FC (s/c)** | **p-value (s-c)** |
| --- | --- | --- | --- | --- |
| **1** | **3310** | **HSPA6** | **198.692** | **0.006** |
| **2** | **18** | **ABAT** | **131.000** | **0.009** |
| **3** | **154197** | **PNLDC1** | **118.857** | **0.001** |
| **4** | **54855** | **TENT5C** | **107.500** | **0.002** |
| **5** | **9074** | **CLDN6** | **93.250** | **0.001** |
| **6** | **441151** | **TMEM151B** | **83.000** | **0.001** |
| **7** | **3398** | **ID2** | **82.054** | **0.005** |
| **8** | **23148** | **NACAD** | **81.500** | **0.005** |
| **9** | **64663** | **SPANXC** | **77.500** | **0.040** |
| **10** | **9935** | **MAFB** | **65.636** | **0.000** |
| **11** | **10371** | **SEMA3A** | **0.046** | **0.002** |
| **12** | **64778** | **FNDC3B** | **0.045** | **0.001** |
| **13** | **6310** | **ATXN1** | **0.044** | **＜0.001** |
| **14** | **23060** | **ZNF609** | **0.044** | **＜0.001** |
| **15** | **1612** | **DAPK1** | **0.042** | **＜0.001** |
| **16** | **169792** | **GLIS3** | **0.042** | **0.001** |
| **17** | **5128** | **CDK17** | **0.041** | **0.001** |
| **18** | **5139** | **PDE3A** | **0.041** | **0.001** |
| **19** | **2887** | **GRB10** | **0.040** | **＜0.001** |
| **20** | **860** | **RUNX2** | **0.033** | **0.003** |

**Table S3**. The differential lipids in Abplatin^(IV)^ treated group (s) compared with the PBS group (c).

FC, fold change; Dark grey: FC≥2; Light grey: FC≤0.5.

|  | **Aberrant lipid** | **RT**  **(min)** | **Quasimolecular Ion** | **m/z** | **Error (ppm)** | **FC (s/c)** | **p-value**  **(s-c)** |
| --- | --- | --- | --- | --- | --- | --- | --- |
| **1** | **LPC(20:3)** | **1.2220** | **[M+H]^+^** | **546.3554** | **0** | **2.4597** | **0.0136** |
| **2** | **LPC(22:5)** | **1.1030** | **[M+H]^+^** | **570.3553** | **0** | **2.5649** | **0.0092** |
| **3** | **LPC(24:0)** | **3.4011** | **[M+H]^+^** | **608.4649** | **0** | **17.5464** | **0.0016** |
| **4** | **LPC(26:0)** | **4.1045** | **[M+H]^+^** | **636.4969** | **1** | **2.2035** | **0.0061** |
| **5** | **LPE(18:0)** | **1.9165** | **[M+H]^+^** | **482.3242** | **0** | **2.9036** | **0.0002** |
| **6** | **LSM(d18:0)** | **1.1088** | **[M+H]^+^** | **467.3608** | **0** | **14.1730** | **0.0010** |
| **7** | **LSM(d20:1)** | **1.6158** | **[M+H]^+^** | **493.377** | **1** | **2.2680** | **0.0109** |
| **8** | **PC(25:0e)** | **3.6563** | **[M+H]^+^** | **622.4815** | **1** | **4.2871** | **0.0016** |
| **9** | **PC(27:0p)** | **3.8227** | **[M+H]^+^** | **648.496** | **0** | **2.1107** | **0.0159** |
| **10** | **PC(30:4e)** | **3.6749** | **[M+H]^+^** | **684.4968** | **1** | **9.8846** | **0.0000** |
| **11** | **PC(12:0p/18:3)** | **3.8070** | **[M+H]^+^** | **684.4963** | **0** | **25.4986** | **0.0003** |
| **12** | **PC(33:7)** | **3.3990** | **[M+H]^+^** | **734.4755** | **0** | **3.2594** | **0.0008** |
| **13** | **PC(33:6)** | **4.1660** | **[M+H]^+^** | **736.4912** | **0** | **2.5063** | **0.0037** |
| **14** | **PC(14:0/24:7)** | **4.2241** | **[M+H]^+^** | **804.5538** | **0** | **3.0230** | **0.0039** |
| **15** | **PC(17:4/22:6)** | **4.3383** | **[M+H]^+^** | **812.5234** | **1** | **2.7787** | **0.0023** |
| **16** | **PC(16:1/23:6)** | **4.5790** | **[M+H]^+^** | **818.5695** | **0** | **2.3576** | **0.0149** |
| **17** | **PC(40:9)** | **3.9680** | **[M+H]^+^** | **828.5553** | **2** | **2.7044** | **0.0118** |
| **18** | **PC(40:7)** | **4.5862** | **[M+H]^+^** | **832.5861** | **1** | **2.9925** | **0.0046** |
| **19** | **PC(24:5/16:1)** | **5.5190** | **[M+H]^+^** | **834.6007** | **0** | **2.5374** | **0.0112** |
| **20** | **PC(42:11)** | **3.7786** | **[M+H]^+^** | **852.5538** | **0** | **3.7734** | **0.0084** |
| **21** | **PC(20:4/22:6)** | **3.9878** | **[M+H]^+^** | **854.5709** | **2** | **3.4368** | **0.0052** |
| **22** | **PC(18:2/24:7)** | **4.4295** | **[M+H]^+^** | **856.5857** | **1** | **3.1380** | **0.0068** |
| **23** | **PC(42:8)** | **4.7589** | **[M+H]^+^** | **858.6013** | **1** | **2.9054** | **0.0068** |
| **24** | **PC(22:6/22:6)** | **4.0066** | **[M+H]^+^** | **878.5706** | **1** | **2.6067** | **0.0079** |
| **25** | **PC(22:5/22:6)** | **4.2035** | **[M+H]^+^** | **880.5867** | **2** | **3.3494** | **0.0096** |
| **26** | **PC(24:5/20:5)** | **4.6846** | **[M+H]^+^** | **882.6007** | **0** | **2.7876** | **0.0178** |
| **27** | **PG(22:6/22:6)** | **3.358** | **[M+H]^+^** | **889.4994** | **0** | **3.8996** | **0.0022** |
| **28** | **PC(18:2/20:4)** | **5.1343** | **[M+HCOO]^-^** | **850.5604** | **0** | **3.9029** | **0.0064** |
| **29** | **PG(20:5/22:6)** | **3.6221** | **[M+H]^-^** | **839.4889** | **2** | **2.5293** | **0.0036** |
| **30** | **PG(22:5/22:6)** | **3.586** | **[M+Na]^+^** | **891.5147** | **0** | **2.5782** | **0.0042** |
| **31** | **SM(d20:1)** | **1.203** | **[M+HCOO]^-^** | **551.3467** | **0** | **2.0186** | **0.0355** |
| **32** | **PC(34:0e)** | **8.8401** | **[M+H]+** | **748.6227** | **2** | **0.3080** | **0.0044** |
| **33** | **PC(36:2e)** | **7.2022** | **[M+H]+** | **772.6218** | **0** | **0.5000** | **0.0005** |
| **34** | **PC(36:1e)** | **8.8984** | **[M+H]+** | **774.6369** | **0** | **0.4178** | **0.0003** |
| **35** | **PC(38:4e)** | **7.0967** | **[M+H]+** | **796.6224** | **1** | **0.5000** | **0.0009** |
| **36** | **PC(38:3e)** | **7.794** | **[M+H]+** | **798.6371** | **0** | **0.5000** | **0.0023** |
| **37** | **PC(38:2e)** | **9.1302** | **[M+H]+** | **800.6537** | **0** | **0.3208** | **0.0000** |
| **38** | **PC(18:0p/22:4)** | **7.0468** | **[M+H]+** | **822.6374** | **0** | **0.5000** | **0.0019** |
| **39** | **PC(40:5e)** | **7.057** | **[M+H]^+^** | **822.6377** | **1** | **0.5000** | **0.0018** |
| **40** | **PC(40:3p)** | **8.2026** | **[M+H]^+^** | **824.6541** | **2** | **0.4275** | **0.0001** |

**Table S4**. The common differential metabolites in Abplatin^(IV)^ treated group (s2), cisplatin treated group (s1) compared with the PBS group (c). RT, retention time; FC, fold change; Dark grey: FC≥2; Light grey: FC≤0.5.

|  | **Aberrant lipid** | **RT**  **(min)** | **Quasimolecular Ion** | **m/z** | **Error (ppm)** | **FC**  **(s1/c)** | **FC (s2/c)** | **p- value**  **(s1-c)** | **p-value**  **(s2- c)** |
| --- | --- | --- | --- | --- | --- | --- | --- | --- | --- |
| **1** | **ATP** | **23.720** | **[M-H]-** | **505.9884** | **0** | **0.2100** | **0.4300** | **0.0027** | **0.0074** |
| **2** | **Nicotinic acid adenine dinucleotide** | **22.854** | **[M+H]+** | **665.0956** | **8** | **0.3000** | **0.2600** | **0.0296** | **0.0271** |
| **3** | **Urocanic acid** | **2.625** | **[M+H]+** | **139.0492** | **7** | **4.1700** | **4.5200** | **0.0269** | **0.0303** |
| **4** | **Inosine** | **6.265** | **[M+H]+** | **269.0864** | **6** | **8.2600** | **12.3000** | **0.0016** | **0.0001** |
| **5** | **Guanosine** | **7.719** | **[M+K]+** | **322.0526** | **7** | **4.2900** | **4.4300** | **0.0159** | **0.0108** |
| **6** | **Glutamate** | **6.095** | **[M+H]+** | **148.0594** | **7** | **7.1400** | **7.1600** | **0.0301** | **0.0105** |
| **7** | **Choline** | **5.298** | **[M+H]+** | **104.1064** | **11** | **4.3000** | **4.7100** | **0.0092** | **0.0126** |
| **8** | **O-glutaroyl-L-carnitine** | **12.706** | **[M+H]+** | **276.1423** | **7** | **4.4600** | **4.8600** | **0.0045** | **0.0030** |
| **9** | **Aspartyl-L-proline** | **15.028** | **[M+H]+** | **231.0961** | **6** | **4.0800** | **3.6500** | **0.0219** | **0.0188** |
| **10** | **Hypoxanthine** | **4.669** | **[M+H]+** | **137.0448** | **7** | **6.3100** | **5.9600** | **0.0006** | **0.0013** |
| **11** | **GSH** | **17.240** | **[M-H]-** | **306.0763** | **1** | **4.9700** | **4.1800** | **0.0008** | **0.1453** |
| **12** | **Cysteine** | **11.900** | **[M-H]^-^** | **120.0122** | **2** | **4.2200** | **3.2100** | **0.0004** | **0.0020** |
| **13** | **Lysine** | **19.968** | **[M+H]^+^** | **147.1117** | **8** | **6.4500** | **3.8300** | **0.0006** | **0.0073** |
| **14** | **Aspartic acid** | **17.278** | **[M+H]^+^** | **134.0439** | **7** | **3.6200** | **2.7400** | **0.0118** | **0.0469** |

**Table S5.** The mainly differential metabolites in Abplatin^(IV)^ treated group (s) compared with the PBS group (c). RT, retention time; FC, fold change; Dark grey: FC≥2; Light grey: FC≤0.5.

|  | **Aberrant metabolites** | **RT**  **(min)** | **Quasimolecular Ion** | **m/z** | **Error (ppm)** | **FC (s/c)** | **p-value**  **(s-c)** |
| --- | --- | --- | --- | --- | --- | --- | --- |
| **Purine metabolism** | **Xanthosine** | **7.873** | **[M+H]+** | **285.0809** | **7** | **3.9113** | **0.0003** |
|  | **Xanthine** | **5.691** | **[M+H]+** | **153.0396** | **7** | **28.8681** | **0.0001** |
|  | **Inosine** | **6.265** | **[M+H]+** | **269.0864** | **6** | **12.3010** | **0.0001** |
|  | **Hypoxanthine** | **6.257** | **[M+H]+** | **137.0448** | **7** | **5.9600** | **0.0013** |
|  | **ATP** | **23.720** | **[M-H]-** | **505.9884** | **0** | **0.4264** | **0.0074** |
| **Arginine biosynthesis** | **Aspartic acid** | **17.278** | **[M+H]+** | **134.0439** | **7** | **2.7436** | **0.0469** |
|  | **Arginine** | **19.210** | **[M+H]+** | **175.1177** | **7** | **41.3248** | **0.0047** |
| **Histidine metabolism** | **Urocanic acid** | **2.635** | **[M+H]+** | **139.0492** | **7** | **4.5165** | **0.0303** |
| **Glutathione metabolism** | **Cysteine** | **11.900** | **[M-H]-** | **120.0122** | **2** | **3.2082** | **0.0020** |
|  | **Glutathione (GSH)** | **17.240** | **[M-H]-** | **306.0763** | **1** | **4.1815** | **0.1453** |
|  | **Glutamate** | **6.095** | **[M+H]+** | **148.0594** | **7** | **7.1596** | **0.0105** |
| **Others** | **Spermidine** | **9.144** | **[M+H]+** | **146.1641** | **7** | **16.3523** | **0.0009** |
|  | **N-Undecanoylglycine** | **1.744** | **[M+H]+** | **244.1890** | **7** | **14.9127** | **0.0016** |
|  | **Lysine** | **19.968** | **[M+H]+** | **147.1117** | **7** | **3.8330** | **0.0073** |
|  | **Homoarginine** | **15.305** | **[M+H]+** | **189.1334** | **6** | **3.6895** | **0.0140** |
|  | **Guanosine** | **9.173** | **[M+K]+** | **322.0526** | **4** | **4.4251** | **0.0108** |
|  | **Guanine** | **7.322** | **[M+H]+** | **152.0557** | **7** | **15.3113** | **0.0416** |
|  | **Tryptophan** | **9.305** | **[M+H]+** | **205.0956** | **8** | **2.5904** | **0.0343** |
|  | **Choline** | **5.298** | **[M+H]+** | **104.1064** | **11** | **4.7053** | **0.0126** |
|  | **Aspartyl-L-proline** | **15.028** | **[M+H]+** | **231.0961** | **6** | **3.6464** | **0.0188** |
|  | **O-glutaroyl-L-carnitine** | **12.706** | **[M+H]+** | **276.1423** | **7** | **4.8600** | **0.0030** |
|  | **Nicotinic acid adenine dinucleotide** | **22.854** | **[M+H]+** | **665.0956** | **8** | **0.2612** | **0.0271** |
|  | **Acetyl-L-histidine** | **13.901** | **[M+H]+** | **198.0859** | **7** | **0.4494** | **0.0042** |
|  | **Carnitine** | **10.246** | **[M+H]+** | **162.1113** | **11** | **0.0317** | **0.0254** |
|  | **Tetradecenoylcarnitine** | **3.238** | **[M+H]+** | **370.2924** | **7** | **0.3299** | **0.0004** |

**References**

1. Chen J, Wang X, Yuan Y, Chen H, Zhang L, Xiao H, Chen J, Zhao Y, Chang J, Guo W, Liang XJ: **Exploiting the acquired vulnerability of cisplatin-resistant tumors with a hypoxia-amplifying DNA repair-inhibiting (HYDRI) nanomedicine.** *Sci Adv* 2021, **7**(eabc5267)**:**1-12.

2. Wang W, Cai J, Wen J, Li X, Yu Y, Zhang L, Han Q, Wei Z, Ma Y, Ying F, et al: **Boosting ferroptosis via abplatin(iv) for treatment of platinum-resistant recurrent ovarian cancer.** *Nano Today* 2022, **44**(101459)**:**1-13.

3. Li X, Li T, Wang Z, Wei J, Liu J, Zhang Y, Zhao Z: **Distribution of perfluorooctane sulfonate in mice and its effect on liver lipidomic.** *Talanta* 2021, **226**(122150)**:**1-11.

4. Wei D, Yu Y, Huang Y, Jiang Y, Zhao Y, Nie Z, Wang F, Ma W, Yu Z, Huang Y, et al: **A Near-Infrared-II Polymer with Tandem Fluorophores Demonstrates Superior Biodegradability for Simultaneous Drug Tracking and Treatment Efficacy Feedback.** *ACS Nano* 2021, **15**(3)**:**5428-5438.

5. Ma J, Wang Q, Yang X, Hao W, Huang Z, Zhang J, Wang X, Wang PG: **Glycosylated platinum(iv) prodrugs demonstrated significant therapeutic efficacy in cancer cells and minimized side-effects.** *Dalton Trans* 2016, **45:**11830-11838.

6. Wang Q, Huang Z, Ma J, Lu X, Zhang L, Wang X, George Wang P: **Design, synthesis and biological evaluation of a novel series of glycosylated platinum(iv) complexes as antitumor agents.** *Dalton Trans* 2016, **45:**10366-10374.
